# Bioactive Secondary Metabolites at the Ends of the Earth (2015–2025): Insights into Arctic and Antarctic Aquatic Sources

**DOI:** 10.3390/md24030093

**Published:** 2026-02-26

**Authors:** Kim-Hoa Phi, Eun Jin Heo, Sunbeom Kwon, Ui Joung Youn, Seulah Lee

**Affiliations:** 1 Division of Life Sciences, Korea Polar Research Institute, Incheon 21990, Republic of Korea; phikimhoa246@gmail.com (K.-H.P.); ujyoun@kopri.re.kr (U.J.Y.); 2 BK21 Interdisciplinary Program in IT-Bio Convergence System, Department of Convergent Biotechnology and Advanced Materials Science, Kyung Hee University, Yongin 17104, Republic of Korea; gjdmswls2389@khu.ac.kr (E.J.H.); sunbeom@khu.ac.kr (S.K.); 3 Center for Space Biomedical Sciences, G-LAMP NEXUS Institute, Kyung Hee University, Yongin 17104, Republic of Korea

**Keywords:** bioactive compounds, secondary metabolites, aquatic sources, Arctic, Antarctica

## Abstract

Marine organisms living in extreme environments such as the Arctic and Antarctic have evolved remarkable adaptation mechanisms to survive harsh conditions, including low temperatures, high salinity, and seasonal fluctuations in light and nutrients. Among these adaptations, unique biochemical pathways have given rise to secondary metabolites with unprecedented chemical structures and diverse biological activities. This review focuses on bioactive natural products that have been isolated from polar aquatic organisms between 2015 and 2025. It provides a comprehensive overview of these compounds, highlighting their chemical structures, source organisms, and documented biological activities. By examining recent discoveries from the ends of the Earth, this review underscores the rich chemical diversity of polar marine ecosystems and their continued potential as a source of novel molecules for drug discovery and biotechnology.

## 1. Introduction

The polar regions, encompassing the Arctic, Antarctica, and their subregions, are among the most remote and environmentally challenging areas on Earth. Living under persistent low temperatures, intense winds, limited nutrient availability, and elevated UV radiation, polar organisms have evolved a remarkable suite of biochemical and physiological adaptations to ensure survival. These adaptations, often driven by shifts in gene regulation and metabolic pathways, increase the likelihood of discovering unique chemical scaffolds and bioactive metabolites of pharmaceutical relevance.

Despite low temperatures and strong seasonality, polar marine environments host complex and dynamic ecosystems comprising diverse groups of microorganisms and fauna, including bacteria, actinomycetes, fungi, algae, sponges, tunicates, and other invertebrates. Natural products from the Arctic or the Antarctic aquatic organisms have been the subject of several review articles: Baker and his group published two comprehensive reviews on cold-water natural products, including compounds from polar marine sources [1,2]. A review on secondary metabolites of polar organisms mentioned compounds derived from polar marine bacteria, sponges, and tunicates [3]. Other reviews on specific groups of compounds, such as terpenoids and meridianins, and mini-reviews on natural products from fungi and bacteria, also mentioned the discovery of secondary metabolites from polar marine environments [4,5,6,7,8,9]. However, to the best of our knowledge, no comprehensive review has been published in the past decade that examines explicitly biologically active compounds from polar marine and aquatic environments. In this review, we compile all reported bioactive secondary metabolites isolated from organisms inhabiting these ecosystems, with particular attention to the biological assays conducted, their biomedical relevance, and the potential significance.

## 2. Bacteria

**Figure 1:**
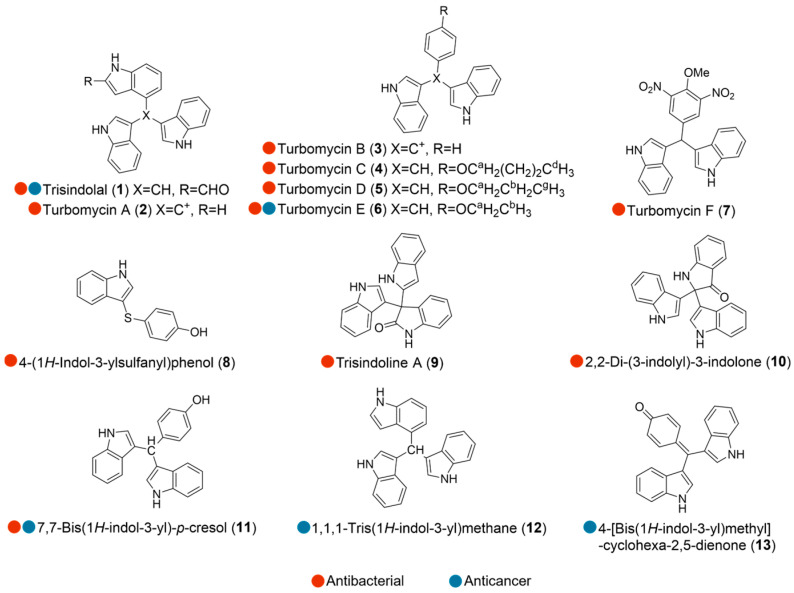
Structures of compounds **1**–**13** and their biological activities.

*Vibrio splendidus* was isolated from the gastrointestinal tract of a fish dredged near the South Orkney Islands in Antarctica. The broth culture of this bacterium yielded a series of new rare indole alkaloids and their known analogues (**1**–**13**, Figure 1) [10]. Among them, trisindolal (**1**) demonstrated excellent cytotoxicity and good tumor selectivity against 11 human tumor cell lines, with individual IC_50_ values ranging from 0.50 µM to 7.82 µM. It also exhibited greater potency than most standard agents, including doxorubicin and paclitaxel. Additionally, trisindolal (**1**) showed broad-spectrum antimicrobial activity against Gram-positive bacteria (*Bacillus subtilis*, *Staphylococcus aureus*, *Streptomyces viridochromogenes*), Gram-negative bacteria (*Escherichia coli*), and fungi (*Candida albicans*, *Mucor miehei*), as well as against several plant-pathogenic fungi, including *Botrytis cinerea* and *Phytophthora infestans*. Other compounds (**2**–**13**) also exhibited anti-microbial and cytotoxic activities (Table 1) [10].

**Figure 2:**
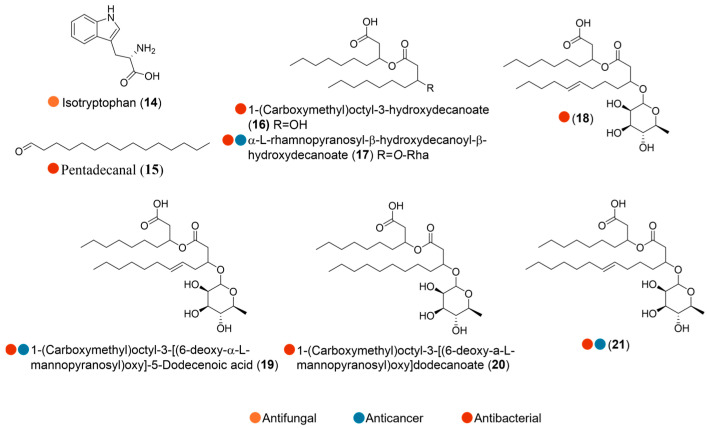
Structures of compounds **14**–**21** and their biological activities.

Isotryptophan (**14**, Figure 2), isolated from the Antarctic seawater bacterium *Bacillus amyloliquefaciens* Pc3, displayed antifungal activity against *Trichoderma viride*, *Colletotrichum gloeosporioides*, *Sclerotinia sclerotiorum*, *Fusarium oxysporum*, *Alternaria longipes*, *Rhizoctonia solani* Kühn, and *Paecilomyces variotii*, with MIC values ranging from 3.125 to 6.25 μg/disc (Table 2). Isotryptophan (**14**), characterized by its thermal stability, emerges as promising candidate for the treatment and prevention of plant-pathogenic fungal infections [11].

Chemical investigation of *Pseudoalteromonas haloplanktis* TAC125, collected from seawater near the French Antarctic Station Dumont d’Urville, led to the isolation of a bioactive compound, pentadecanal (**15**, Figure 2). This long-chain fatty aldehyde acted as a signaling molecule, effectively inhibiting biofilm formation of *Staphylococcus epidermidis*—a major factor in medical device-related infections that often exhibits resistance to conventional antibiotics (Table 2). Interestingly, the compound showed no bacteriostatic or bactericidal activity up to the highest tested concentration of 1.6 mg/mL. This characteristic indicates high specificity of action, helping reduce the risk of resistance and giving potential in vivo application when pairing with conventional antibiotics [12].

Arctic marine bacterium *Pseudomonas* sp. M10B774 was collected from an Atlantic halibut in the Norwegian Sea. One new and four known mono-rhamnolipids, as well as the lipid moiety from one of the rhamnolipids (**16**–**21**, Figure 2), were successfully obtained through bioactivity-guided isolation combined with MS/MS-based molecular networking for dereplication. These compounds showed weak to moderate antibacterial activity against three Gram-positive bacteria, *Enterococcus faecalis*, *Staphylococcus aureus*, and *Streptococcus agalactiae*. Additionally, compounds **16** and **17** exhibited strong anti-biofilm activity at 50 µM, whereas the others showed activity at 100 µM. Compounds **17**, **19**, and **21** showed weak cytotoxicity against the human melanoma cell line A2508 and the non-malignant MRC5 cell line. These findings suggest that the *Pseudomonas* sp. strain used in this study could be a candidate to replace *Pseudomonas aeruginosa* for industrial production of rhamnolipids, helping reduce the risk due to human pathogenicity of this bacterium (Table 2) [13].

**Figure 3:**
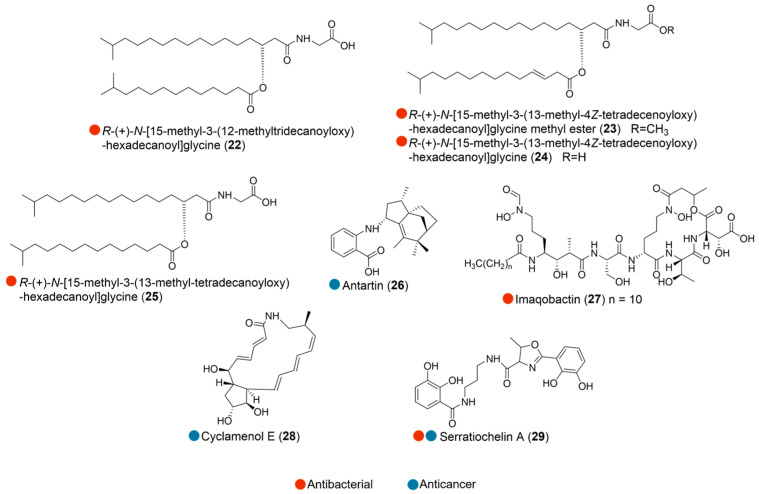
Structures of compounds **22**–**29** and their biological activities.

The combination of an LC-MS/MS-based metabolomics strategy and an anti-MRSA (methicillin-resistant *Staphylococcus aureus*) activity-guided fractionation scheme was applied to the Gram-negative bacterium *Aequorivita* sp., isolated from shallow Antarctic Sea sediment. This approach led to the discovery of linear aminolipids bearing an *N*-terminal glycine unit (**22**–**25**, Figure 3) that showed moderate in vitro antimicrobial activity against MRSA (Table 3). In comparison with non-bioactive analogs, glycine appears to be the favored *N*-terminal unit for antibacterial activity, while the introduction of a double bond or methylation of the iso-fatty acid chain could diminish or totally abolish antibiotic activity [14].

Actinomycete *Streptomyces* sp. was isolated from marine sediments collected off the coast of Antarctica. Its culture broth yielded antartin (**26**, Figure 3), a new zizaane-type sesquiterpene displaying potent cytotoxicity against various cancer cell lines at 20 µg/mL. Further studies revealed that this compound suppressed the proliferation of lung cancer cells (A549, H1299) and brain tumor cells (U87) by inducing G1 cell cycle arrest (Table 3) [15].

Imaqobactin (**27**, Figure 3) was isolated as a new compound from *Variovorax* sp. RKJM285, found in marine sediment near Clyde River, Nunavut, Canada. It exhibited moderate activity against MRSA, vancomycin-resistant *Enterococcus*, *Staphylococcus warneri*, and *Proteus vulgaris*, with IC_50_ ranging from 11 to 35 μM. This activity is likely due to the ability of imaqobactin to bind Fe(III), thereby inhibiting microbial proliferation by depleting cellular iron (Table 3) [16].

*Streptomyces* sp. OUCMDZ-4348 was isolated from a sand sample collected in Antarctica. The liquid fermentation yielded a new bicyclic macrolactam, cyclamenol E (**28**, Figure 3). The cytotoxicity of **28** against a panel of 26 cancer cell lines and 2 normal cells was tested, and it was found to inhibit the gastric carcinoma cell line N87 with high selectivity. No cytotoxic effect was observed against other cell lines, suggesting the high selectivity of cyclamenol E and providing direction for the study of anticancer mechanisms (Table 3) [17].

A siderophore isolated from a co-culture of the Atlantic hagfish digestive tract-derived *Serratia* sp. and *Shewanella* sp., serratiochelin A (**29**, Figure 3), selectively inhibited *Staphylococcus aureus* growth with an MIC of 25 μM. Additionally, **29** also reduced the cell proliferation of both eukaryotic cell lines tested, the human melanoma cell line A2058 and the non-malignant lung fibroblast cell line MRC5. It also inhibited biofilm formation of *Staphylococcus epidermidis* (Table 3) [18].

**Figure 4:**
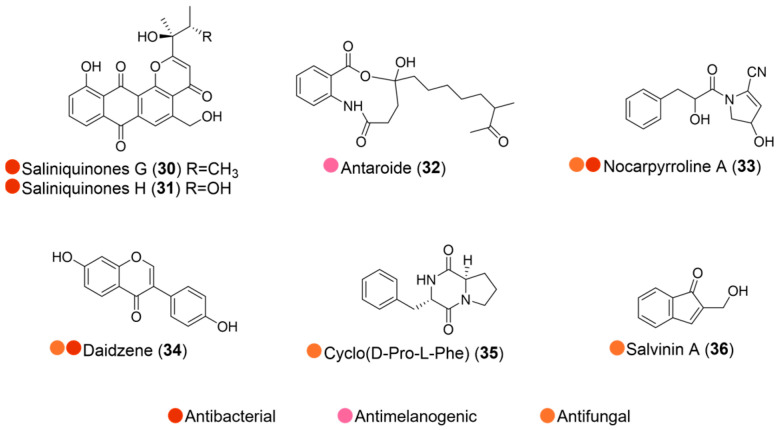
Structures of compounds **30**–**36** and their biological activities.

Two new anthraquinone derivatives, saliniquinones G and H (**30** and **31**, Figure 4), were obtained from the Antarctic marine animal-derived actinomycete *Nocardiopsis aegyptia* HDN19-252. They showed promising inhibitory activity against six bacterial strains, including methicillin-resistant coagulase-negative *staphylococci* (MRCNS), with MIC values ranging from 3.1 to 12.5 µM, which were stronger than those of the positive control, ciprofloxacin (50 µM) (Table 4). In particular, their activities against MRCNS were 8-fold stronger than ciprofloxacil, highlighting the potential for screening and developing antibiotics from Actinomycete-derived saliniquinones [19].

Antaroide (**32**, Figure 4), a new nine-membered macrolide isolated from the Antarctic marine sediments-derived bacterium *Streptomyces* sp. SCO-736, demonstrated an anti-melanogenic activity by suppressing the mRNA expression of the melanogenic enzymes such as tyrosinase, TRP-1, and TRP-2 (Table 4) [20].

The Actinomycetes *Nocardiopsis* sp. LX-1, derived from the Antarctic krill *Euphausia superba*, produced several compounds (**33**–**36**, Figure 4) with weak anti-microbial activity. Nocarpyrroline A (**33**) was active against *Fusarium fujikuroi* and *Aeromonas hydrophila*. Daizene (**34**) showed broad antibacterial activity against *A. hydrophila*, *Dickeya chrysanthemi*, *Comamonas terrigena*, and *Xanthomonas citri pv. malvacearum*, with MIC values ranging from 25 to 100 µM. Additionally, all these isolates also showed antifungal activity; nocarpyrroline A (**33**) was active against *F. fujikuroi*, daizene (**34**) was active against *C. albicans*, cyclo(D-Pro-L-Phe) (**35**) and salvinin A (**36**) were active against *D. citri* (Table 4) [21].

**Figure 5:**
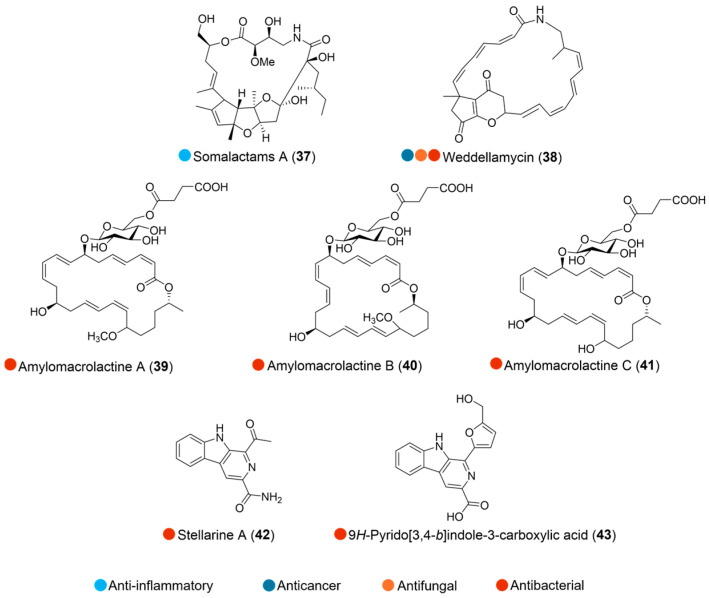
Structures of compounds **37**–**43** and their biological activities.

A strain *Streptomyces somaliensis* 1107, isolated from an Arctic *Haliclona* sponge, yielded a new bioactive somalactam A (**37**, Figure 5), a macrolactam featuring unique ring systems and potent anti-inflammatory activity without cytotoxicity. Treatment with **37** suppressed the production of IL-6 and TNF-α in the culture media of LPS-stimulated RAW264.7 macrophage cells, modulated the MAPK pathway, and alleviated LPS-induced systemic inflammation in a transgenic fluorescent zebrafish model (Table 5) [22].

Weddellamycin (**38**, Figure 5), a new tricyclic polyene macrolactam from *Streptomyces* sp. DSS69 (derived from an Antarctic deep-sea marine sponge collected in the Weddell Sea), exhibited potent activity against *Staphylococcus aureus*, MRSA, methicillin-resistant *Staphylococcus epidermidis* (MRSE), *Enterococcus faecalis*, *Micrococcus luteus*, *Bacillus altitudinis*, *Listeria monocytogenes*, and *Candida albicans* (MICs from 0.10 to 3.33 µg/mL). Additionally, it also showed potent cytotoxicity against human cancer cell lines, including leukemia HL-60, hepatoma HepG2, glioblastoma U-87MG, and colon cancer HCT116 (IC_50_ = 2.07–11.50 µM) (Table 5) [23].

The bacterial strain *Bacillus amyloliquefaciens* SCSIO 41392 was isolated from deep-sea sediments at depths of over 2000 m in the Arctic Ocean. The investigation on its large-scale fermentation led to the isolation of three new 24-membered macrolactins, amylomacrolactines A–C (**39**–**41**, Figure 5, Table 5), along with two known compounds, stellarine A and 9*H*-pyrido [3,4-b]indole-3-carboxylic acid (**42** and **43**, Figure 5, Table 5). The biological evaluation focused on inhibiting various virulence phenotypes of *Pseudomonas aeruginosa*, revealing that all isolated compounds displayed multiple bacterial virulence inhibition activities. Specifically, compounds **39** and **40** exhibited quorum-sensing (QS) inhibitory activity against the PQS system and suppressed the synthesis of the PQS-regulated virulence factor pyocyanin. Compounds **41**–**43** efficiently inhibited pyoverdine production, an essential virulence factor. Furthermore, compound **43** demonstrated efficient anti-biofilm activity against *P. aeruginosa* (Table 5). A structure–activity relationship (SAR) study suggested that the hydroxy group at C-15 might play an essential role in the antimicrobial activity of these isolates [24].

Across diverse polar bacteria, numerous bioactive compounds with structurally diverse scaffolds have been identified. Antimicrobial activity predominates among the reported bioactivities, highlighting polar bacterial as a promising reservoir for anti-infective molecules discovery. However, most studies continue to rely on classical strain isolation from host organisms or sediments, followed by fermentation and in vitro assays, with limited incorporation of genome mining, biosynthetic gene clusters (BGCs), or ecological hypothesis-driven discovery strategies. Future investigations should prioritize the integration of metabolomics-guided dereplication and targeted BGC analysis with mechanistic infection models. This synergistic approach will minimize redundant discovery and more precisely correlate polar bacterial metabolites with clinically significant targets.

## 3. Fungi

**Figure 6:**
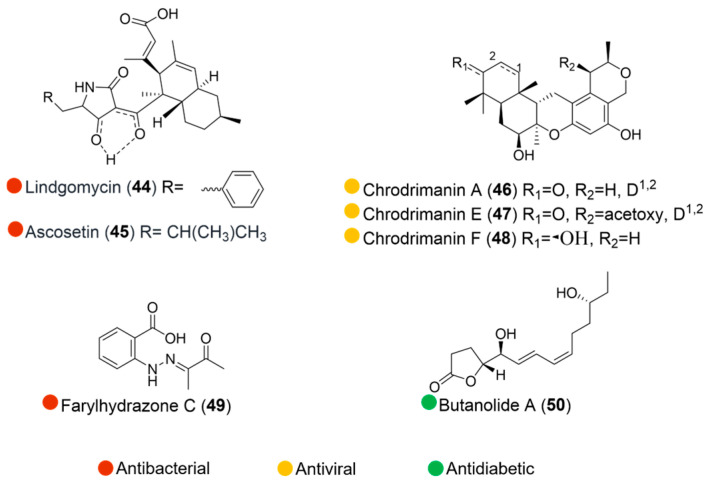
Structures of compounds **44**–**50** and their biological activities.

Lindgomycin and ascosetin (**44** and **45**, Figure 6), unusual polyketides, were extracted from mycelia and culture broth of different Lindgomycetaceae strains collected in the Arctic and Baltic Sea. Both compounds exhibited strong inhibitory activities against fungi and Gram-positive bacteria, with IC50 values ranging from 2 to 18 μM. The effects against the clinically relevant bacteria *Staphylococcus epidermidis*, *Staphylococcus aureus*, MRSA, and *Propionibacterium acnes* were two times less in comparison with chloramphenicol. However, no effect was observed on Gram-negative strains *Escherichia coli* and *Pseudomonas aeruginosa* (Table 6) [25].

The fungal strain *Penicillium funiculosum* GWT2- 24, isolated from moss collected around the China Great Wall Station in Antarctica, produced three biologically active meroterpenoids chrodrimanins A, E, and F (**46**–**48**, Figure 6). These compounds showed inhibitory activities against influenza virus A (H1N1), with IC_50_ values ranging from 21 to 57 μM, which were stronger than the positive control ribavirin (Table 6) [26].

Fermentation broth of the Antarctic-derived fungus *Penicillium* sp. HDN14-431, isolated from the soil of the meso littoral zone, yielded a rare phenylhydrazone, farylhydrazone C (**49**, Figure 6). The compound showed an inhibitory effect against the bacterium *Proteus vulgaris*, with an MIC of 22.5 μM (Table 6) [27].

The fungus *Penicillium* sp. S-1-18 was isolated from the Antarctic seabed sediments. Under the bioassay guidance, a new furanone derivative, butanolide A (**50**, Figure 6), was obtained and showed moderate inhibitory activity against protein tyrosine phosphatase 1B, with an IC_50_ value of 27.4 μM (Table 6) [28].

**Figure 7:**
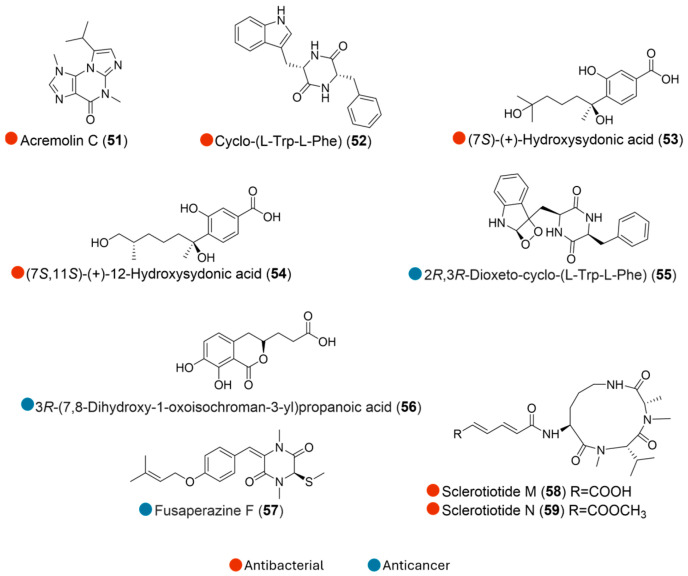
Structures of compounds **51**–**59** and their biological activities.

The fungus *Aspergillus sydowii* SP-1 was isolated from a marine sediment sample collected at the Antarctic Great Wall Station. Its chemical investigation led to the isolation of four compounds (**51**–**54**, Figure 7), including a new alkaloid, acremolin C (**51**), as well as cyclo-(L-Trp-L-Phe) (**52**), hydroxysydonic acid (**53**), and its derivative (**54**). These compounds exhibited weak to moderate inhibitions against MRSA and MRSE, with MIC values ranging from 0.5 to 32 μg (Table 7) [29].

Chemical investigation on the static culture of Antarctic fungus *Penicillium citreonigrum* SP-6 collected initially at the site of Antarctic Great Wall Station led to the discovery of a diketopiperazine and a phenol (**55** and **56**, Figure 7). These inhibited the human colon cancer cell line HCT116, with IC_50_ values of 26.7 and 46.3 μM, respectively (Table 7). Considering the cytotoxic activities of the two compounds, the 1, 2-dioxetane radical group in compound **55** is likely to elevate the cytotoxic activities of the diketopiperazine [30].

A new diketopiperazine (**57**, Figure 7), isolated from the Antarctic fungus *Penicillium crustosum* HDN153086 (collected in Prydz Bay), exhibited cytotoxicity against lymphoblast cells K562, with an IC_50_ value of 12.7 μM (Table 7). Comparing the structure of **57** with non-bioactive analogs suggested that the double bond between C-6 and C-7 might contribute to its activity, while substitution at C-6 might eliminate the effect on K562 cells [31].

*Aspergillus insulicola* HDN151418 was isolated from an unidentified sponge sample collected 410 m deep from Prydz Bay, Antarctica. From its static culture, two new bioactive aspochracin-type cyclic tripeptides, sclerotiotides M and N (**58** and **59**, Figure 7), were discovered. These isolates showed broad antimicrobial activity against a panel of pathogenic strains, including *Bacillus cereus*, *Proteus species*, *Mycobacterium phlei*, *B. subtilis*, *Vibrio parahemolyticus*, *Edwardsiella tarda*, MRCNS, and MRSA, with MIC values ranging from 1.56 to 25.0 µM. Notably, these two compounds showed potent activity against *M. phlei*, which provides potential candidates for antitubercular drug development (Table 7) [32].

**Figure 8:**
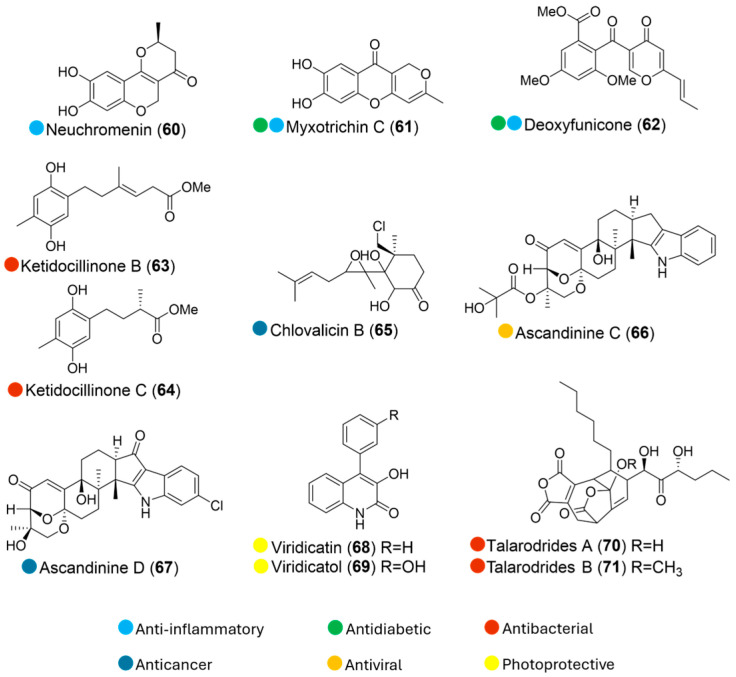
Structures of compounds **60**–**71** and their biological activities.

Antarctic marine-derived fungal strain *Penicillium glabrum* SF-7123 was isolated from sediments collected at the Ross Sea. Isolation of its fermented culture led to the discovery of neuchromenin, myxotrichin C, and deoxyfunicone (**60**–**62**, Figure 8). These compounds exhibited anti-inflammatory activity without cytotoxicity in LPS-stimulated BV2 and RAW264.7 cells by inhibiting excessive nitric oxide (NO) production and LPS-induced overproduction of prostaglandin E2 in both cellular models (Table 8). Further mechanistic studies revealed that the most active compound, **60**, can significantly suppress the overexpression of inducible nitric oxide synthase and cyclooxygenase-2 at a concentration of 4 µM, accompanied by downregulation of inflammation-related signaling pathways. Structure–activity comparison between **60** and its inactive analogue suggests that replacement of the hydroxy group at the C-9 position with a methoxy group markedly diminishes anti-inflammatory activity. In addition, compound **61** was identified as a noncompetitive inhibitor of PTP1B, with an IC_50_ value of 19.2 µM, and compound **62** was shown to inhibit the activity of PTP1B, with an IC_50_ value of 24.3 µM, by binding to the active site of the enzyme [33].

Two new polyketides were isolated from the Antarctic sponge-derived fungus *Penicillium* sp. HDN151272 (collected at Prydz Bay, depth 410 m). Ketidocillinones B and C (**63** and **64**, Figure 8) exhibited potent antibacterial activity against *Pseudomonas aeurigenosa*, *Mycobacterium phlei*, and MRCNS, with MIC values ranging from 1.56 to 25 µg/mL (Table 8). Through structure–activity comparison between all isolates, the methoxy group was suggested to play a crucial role in the anti-bacterial activity [34].

The marine fungus *Digitatispora marina* was isolated from the driftwood of the *Betula* sp. collected at Vanna, Norway. A new chlorinated metabolite, chlovalicin B (**65**, Figure 8), was isolated from liquid cultures of the fungus, exhibiting weak cytotoxic activity against the human melanoma cell line A2058 (Table 8) [35].

The fungal strain *Aspergillus candidus* HDN15-152 was isolated from the sponge collected from Pulitzer Bay, Antarctica. Its chemical investigation led to the isolation of two new bioactive indole diterpenoids, ascandinines C and D (**66** and **67**, Figure 8). Ascandinine C (**66**) displayed anti-influenza virus A (H1N1) activity, while ascandinine D (**67**) showed cytotoxicity against the human leukemia cell line HL-60 (Table 8) [36].

The fungal strain *Penicillium echinulatum* was isolated as an endophyte from the fresh surface-sterilized tissue of the brown alga *Adenocystis utricularis* collected at Ipanema beach, King George Island, Antarctica. Viridicatin and viridicatol (**68** and **69**, Figure 8), isolated from its static fermented culture, showed significant UV and were considered photostable after UVA irradiation (Table 8). Their critical wavelengths (λ_c_) represent an intermediate level of UVA protection, similar to the commercial UV filter benzophenone-3. No phototoxicity was observed in the RHS model, and both compounds inhibited UVA-induced ROS generation in HaCaT cells. These results indicate their low acute photo irritation and high photo safety potential in humans. Together with their photoprotective and antioxidant potential, these compounds can be considered a new class of molecules for photoprotection, since their photo safety and non-cytotoxicity were predicted using recommended in vitro methods for topical use [37].

Two new nonadride derivatives isolated from the Antarctic sponge-derived fungus *Talaromyces* sp. HDN1820200, talarodrides A and B (**70** and **71**, Figure 8), showed selective inhibitory effects against *Proteus mirabilis* and *Vibrio parahemolyticus* with MICs of 3.13–12.5 μM (Table 8). Among these, **71** displayed stronger inhibition against *P. mirabilis* and *V. parahemolyticus* than **70**, which indicated that the methoxy group may play a key role in the antibacterial effect [38].

**Figure 9:**
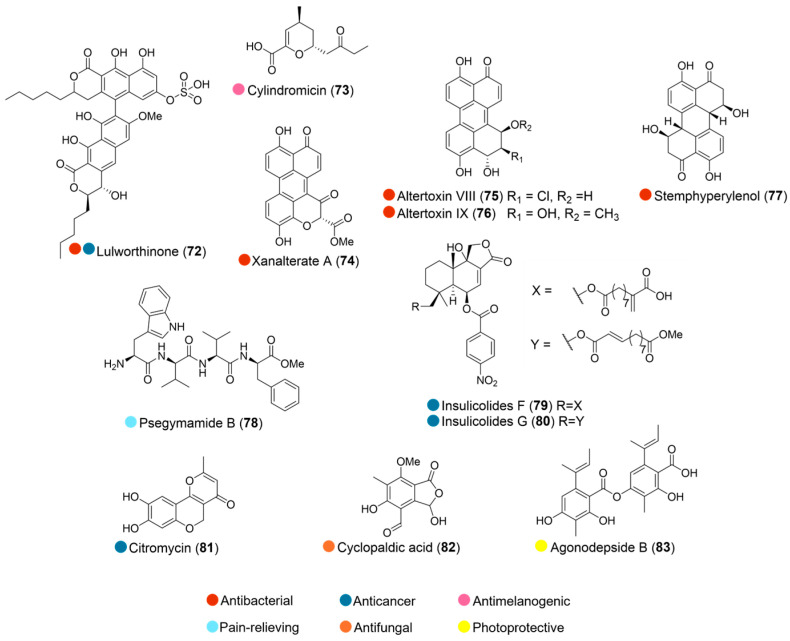
Structures of compounds **72**–**83** and their biological activities.

The marine fungus 067bN1.2, belonging to Lulworthiaceae, was isolated from a dead pine (*Pinus* sp.) collected in the splash zone in Kongsfjord, Berlevåg, Norway. Through bioactivity-guided isolation, lulworthinone (**72**, Figure 9), a new dimeric naphthopyrone, was discovered and showed antibacterial activity against *Staphylococcus aureus*, *Streptococcus agalactiae*, and several clinical MRSA strains with MICs in the 1.56–6.25 μg/mL range. It also exhibited antiproliferative activity against human melanoma, hepatocellular carcinoma, and non-malignant lung fibroblast cell lines (Table 9). Further study revealed that lulworthinone induces the upregulation of cell envelope stress-response genes in *Bacillus subtilis*, modulating bacterial membrane function while maintaining structural integrity [39,40].

A new compound, cylindromicin (**73**, Figure 9), isolated from Arctic glacier sediment-derived fungus *Tolypocladium* sp. SCSIO 40433, exhibited significant tyrosinase inhibition activity from 20 µM (Table 9). SAR studies suggested that the carboxyl group at C-6 and carbonyl group at C-9 of **73** played important roles in the tyrosinase inhibition activity [41].

Three perylenequinone derivatives, namely xanalterate A, altertoxin VIII and IX, together with one known analogue, stemphyperylenol (**74**–**77**, Figure 9), were isolated from the extract of the Antarctic sponge-derived fungus *Alternaria* sp. HDN19-690. All the isolates exhibited broad antibacterial activity, with compound **74** the best (MIC values ranging from 3.13 to 12.5 µM) (Table 9) [42].

The fungal strain *Pseudogymnoascus* sp. HDN17-933 was isolated from Fildes Peninsula, Antarctica. A new tetrapeptide psegymamide B (**78**, Figure 9), isolated from its fermentation broth, showed significant inhibitory activity on human nicotinic acetylcholine receptor subtypes (Table 9). A preliminary SAR investigation revealed that the tryptophan residue and the *C*-terminal with a methoxy group were essential to the inhibitory activity [43].

Two new nitrobenzoyl sesquiterpenoids, insulicolides F and G (**79** and **80**, Figure 9), were isolated from the sponge-derived fungus *Aspergillus insulicola* HDN151418 (collected from Prydz Bay, Antarctica). Both showed selective inhibition against human pancreatic ductal adenocarcinoma (PDAC) cell lines (Table 9). Mechanistic studies revealed that insulicolide G (**80**) suppressed PDAC cell proliferation, induced apoptosis, and blocked migration and invasion. This compound could also prevent resistance and enhance the therapeutic effect of the chemotherapy drug gemcitabine in PDAC cancer [44].

Citromycin (**81**, Figure 9), isolated from the Antarctic marine-derived fungus *Sporothrix* sp., inhibited the migration and invasion of human ovarian cancer SKOV3 and A2780 cells, but had no cytotoxic activity against them. Mechanistic investigation suggested that citromycin exhibits its anticancer activity on human ovarian cancer by downregulating the expression levels of EMT markers and MMP-2/9 via inhibition of the ERK1/2 pathway [45].

The fungus *Penicillium* sp. strain CRM 1540 was isolated from marine sediment collected at Admiralty Bay (King George Island, Antarctica). Cyclopaldic acid (**82**, Figure 9), obtained from this strain, revealed potential as a leading molecule against phytopathogenic fungi of global agricultural importance, *Macrophomina phaseolina* and *Rhizoctonia solani*, with more than 90% of growth inhibition after 96 h of contact with the fungal cells using a concentration of 100 µg/mL, and more than 70% using 50 µg/mL [46].

Agonodepside B (**83**, Figure 9), isolated from Antarctic fungus *Arthrinium* sp., was able to protect viable epidermis against UVA-induced ROS production, both in keratinocyte monolayers and in reconstructed human skin models, with a reduction of 30.2% in the fluorescence in 3D skin models. It did not present any phototoxic potential, was demonstrated to be photostable and non-cytotoxic to HaCaT cells, and was classified as a slight irritant in the HETCAM assay. These results suggested that it could be a promising antioxidant and photoprotective agent [47].

**Figure 10:**
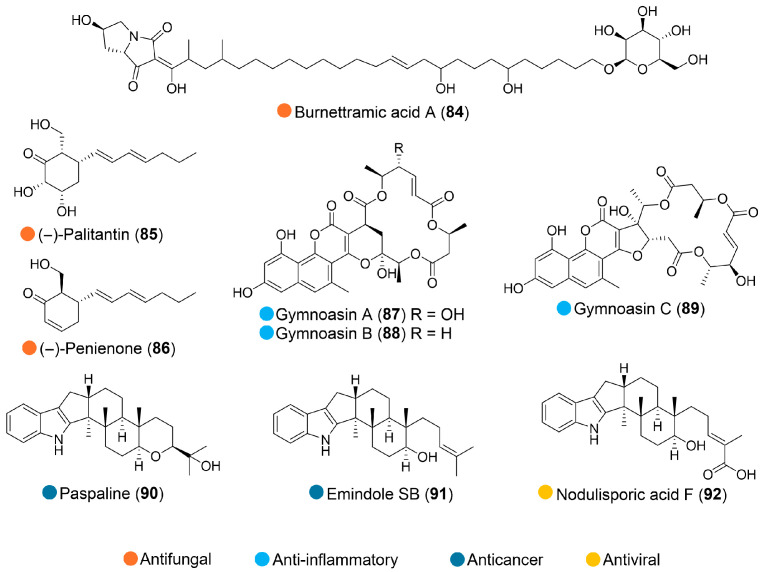
Structures of compounds **84**–**92** and their biological activities.

Burnettramic acid A (**84**, Figure 10), isolated from the arctic deep-sea fungus *Aspergillus versicolor* PS108-62, inhibited the growth of *Candida albicans* with an IC_50_ value of 7.2 µg/mL [48].

The fungus *Penicillium palitans* (Ascomycota) was collected from deep-sea marine sediment samples obtained at 404 m depth in the Southern Ocean, maritime Antarctica. Two bioactive isolates were isolated. (–)-Palitantin (**85**, Figure 10) exhibited moderate activity on the growth of *Lactuca sativa* and *Agrostis stolonifera* seedlings and no antifungal activity. (–)-Penienone (**86**, Figure 10) demonstrated phytotoxicity against *Lactuca sativa*, *Agrostis stolonifera*, and significant antifungal activity against *Colletotrichum fragariae (*Table 10). These biological profiles position these compounds as promising candidates for the development of novel agrochemicals, warranting further investigation [49].

The fungal strain *Pseudogymnoascus* sp. HDN17-895 was isolated from a soil sample collected in the intertidal zone of the Fildes Peninsula, Antarctica. Scale-up fermentation yielded three novel naphthopyrone–macrolide hybrids with unusual chemical architectures, gymnoasins A–C (**87**–**89**, Figure 10). These compounds dose-dependently inhibit LPS/ATP-induced IL-1β release and **87** demonstrated effective, selective anti-inflammatory activity in vivo (Table 10). Specifically, it significantly inhibited in vitro NLRP3 inflammasome activation and in vivo pro-inflammatory cytokine IL-1β release, representing a valuable new lead compound for the development of novel anti-inflammatory agents [50].

From the Antarctic sponge-derived fungus *Aspergillus candidus* HDN15-152 (collected from Pulitzer Bay), paspaline, emindole SB, and nodulisporic acid F (**90**–**92**, Figure 10) were isolated. **90** and **91** demonstrated significant cytotoxic activity against several cell lines (NCI-H446, NCI-H446/EP, and L-02) while **92** displayed antiviral activity against the influenza A virus (IAV) A/PR/8/34(H1N1) strain, with an IC_50_ value comparable to that of the positive control ribavirin (Table 10) [51].

**Figure 11:**
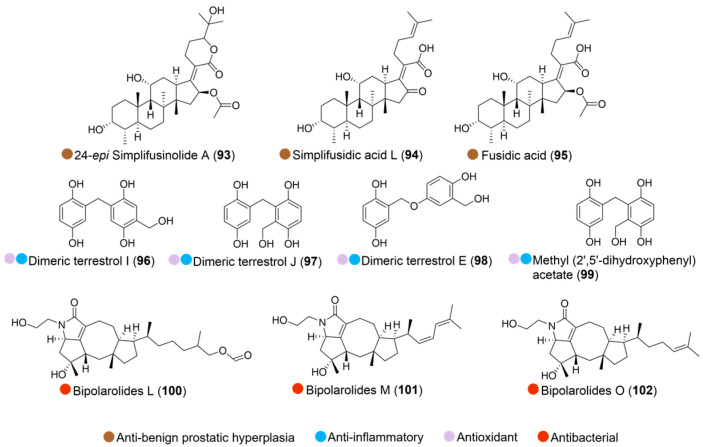
Structures of compounds **93**–**102** and their biological activities.

Two new metabolites, 24-*epi* simplifusinolide A and simplifusidic acid L, were isolated, along with fusidic acid (**93**–**95**, Figure 11), from the Arctic marine-derived fungus *Simplicillium lamellicola* (collected from seawater around the Dasan Korean Arctic Station in Ny-Alesund, Svalbard). These compounds demonstrated significant anti-benign prostatic hyperplasia (BPH) effects by targeting the androgen receptor signaling pathway, suggesting them to be potential alternatives for BPH treatment (Table 11) [52].

Two new gentisyl alcohol derivatives, dimeric terrestrol I and J, together with two known compounds (**96**–**99**, Figure 11), were isolated from the Arctic surface seawater-derived fungus *Aspergillus japonicas*. All four compounds showed strong in vitro antioxidant activity, comparable with vitamin C. Terrestrol J (**97**) showed strong in vitro anti-inflammatory activity, even stronger than dexamethasone (Table 11). It alleviated lipopolysaccharide-induced BV2 microglial cell death by reducing nitric oxide production. The activity difference of **96** and **97** was possibly caused by a hydroxy methyl group attached to C-6′ instead of C-4′ [53].

The fungus *Uzbekistanica storfjordensis* sp. nov was isolated from deciduous wood collected from the intertidal zone of Taterneset, Storfjord municipality, Troms, Norway. Three new sesterterpenes, bipolarolides L, M, and O (**100**–**102**, Figure 11), derived from the fungus, exhibited inhibitory activity against *Streptococcus agalactiae (*Table 11). Structure–activity relationship analysis revealed that the presence of two methyl substituents at C-25 and a hydroxylated ethylene substituent at N-3 seems important to the antibacterial effect [54].

**Figure 12:**
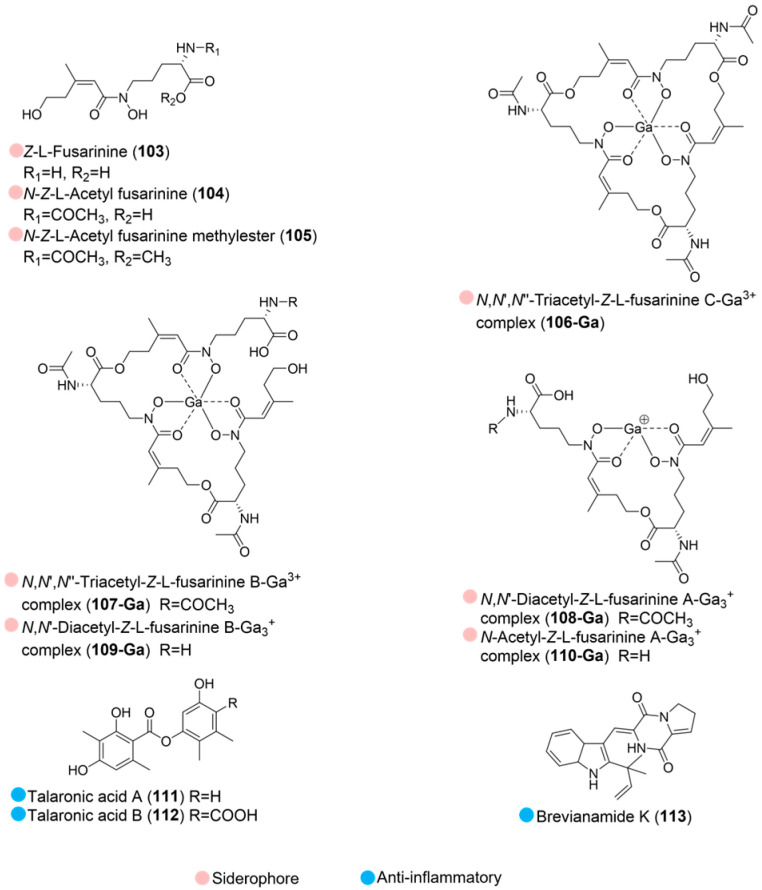
Structures of compounds **103**–**113** and their biological activities.

Eight fusarinine-type hydroxamates (**103**–**110**, Figure 12) were isolated from Antarctic sponge-derived fungus *Pseudogymnoascus verrucosus* under iron-deficient conditions. All of these compounds exhibited siderophore activity, effectively promoting fungal growth. Furthermore, gallium-chelated forms of compounds **103**–**107** also supported fungal growth and exerted fungistatic effects, likely due to their interference with iron homeostasis within the cell [55].

Two new depsides, talaronic acids A and B (**111** and **112**, Figure 12), were isolated from *Talaromyces* sp. HDN1820200 (collected in the Antarctic Weddell Sea, at a depth of 346 m) and exhibited anti-inflammatory activity at 5 µM. This result aligns with previous studies on the remarkable anti-inflammatory effect of structurally related depsides, suggesting hetero dimer depsides as a promising source for novel anti-inflammatory agents [56].

*Aspergillus* sp. (strain SF7367) was isolated from calcareous algae collected at the Barton Peninsula, Antarctica. Brevianamide K (**113**, Figure 12) was isolated from the strain, which exhibited anti-inflammatory effects in both lipopolysaccharide-stimulated BV2 microglia and RAW264.7 macrophages (Table 12). In vitro and in silico studies revealed that this compound exerts its ability by reducing lipopolysaccharide-induced nuclear translocation of NF-κB (p65) [57].

Polar fungi, occurring both as free-living organisms and in association with algae, sediments, driftwood, and sponges, yield structurally diverse polyketides, peptides, meroterpenoids, siderophores, and indole diterpenoids with antibacterial, antiviral, antitubercular, anti-inflammatory, photoprotective, and enzyme-inhibitory activities. These findings underscore fungi as a rich and versatile source of pharmacologically relevant metabolites. However, like bacterial studies, fungal investigations are predominantly culture-based, focusing on easily cultivable genera such as *Penicillium* and *Aspergillus*, and often terminating at preliminary in vitro bioassays with limited mechanistic insights or SAR analysis. Broader integration of metagenomics, co-culture strategies, epigenetic modulation, and untargeted metabolomics will be essential to unlock the hidden metabolome of uncultured or low-producing strains and to rationally prioritize fungal metabolites with improved selectivity, bioavailability, and safety profiles.

## 4. Sponge

**Figure 13:**
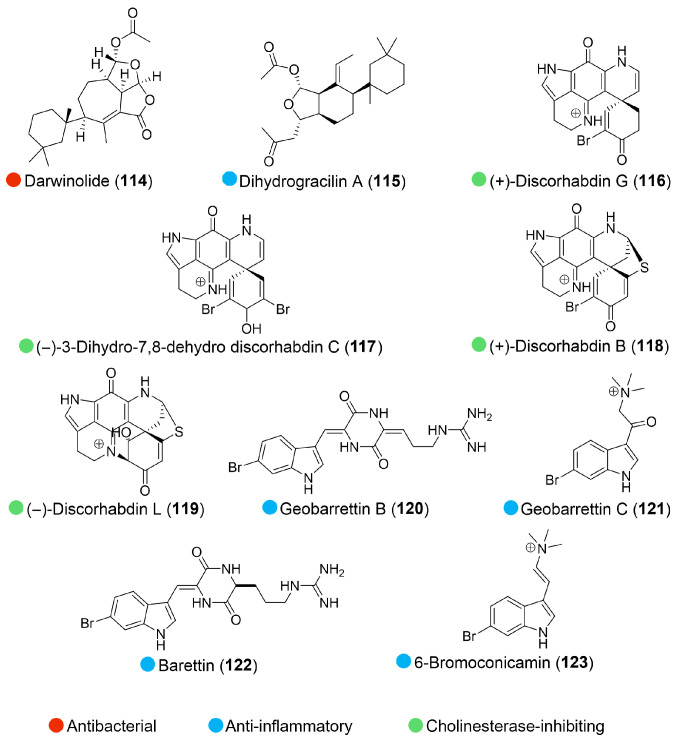
Structures of compounds **114**–**123** and their biological activities.

A compound with a new diterpene scaffold, darwinolide (**114**, Figure 13), was isolated from the Antarctic sponge *Dendrilla membranosa* (obtained from the vicinity of Palmer Station). This compound displayed 4-fold selectivity against MRSA biofilm over planktonic cells, along with low mammalian cytotoxicity, suggesting that it could serve as a highly suitable scaffold for the development of novel antibiofilm-specific antibiotics (Table 13) [58].

Dihydrogracilin A (DHG, **115**, Figure 13), a diterpenoid from the Antarctic marine sponge *Dendrilla membranosa,* demonstrated significant immunomodulatory and anti-inflammatory properties across various models. In vitro, DHG decreased the proliferation and viability of activated human immune cells (PBMC) by inducing apoptosis and downregulating key inflammatory signaling pathways. Furthermore, it reduced the growth and migration of human keratinocytes. And most significantly, DHG demonstrated strong topical anti-edema activity in a murine dermatitis model, suggesting its potential use as an anti-inflammatory agent for cutaneous diseases (Table 13) [59].

Discorhabdin alkaloids (**116**–**119**, Figure 13) were isolated from Antarctic *Latrunculia* spp. sponge (dredged from coastal shelf environments around the Antarctic Peninsula, at depths between 200 m and 600 m). These compounds were reported as reversible competitive inhibitors of cholinesterase, with inhibitory constants (*K_i_*) ranging from 1.6 µM against *Electrophorus electricus* AChE to 98 µM against human AChE, suggesting their therapeutic potential for Alzheimer′s disease (Table 13). Notably, electrophysiological experiments confirmed that the most potent analogue, discorhabdin G (**116**), exhibited no detectable adverse effects on neuromuscular transmission or skeletal muscle function. This suggests a favorable safety profile that may mitigate the peripheral side effects typically associated with conventional cholinesterase inhibitors [60].

Two new bromoindole alkaloids, geobarrettins B and C, together with two known compounds (**120**–**123**, Figure 13), were isolated from the Icelandic marine sponge *Geodia barretti* (collected in the west of Iceland at 388 m depth), which exhibited promising anti-inflammatory characteristics. Geobarrettin B (**120**) and C (**121**) primarily exerted their effects by reducing the inflammatory Th1 response, which is associated with numerous chronic inflammatory diseases, while barettin (**122**) potently inhibited both IL-12p40 and IL-10 secretion by DCs in a dose-dependent manner, and 6-bromoconicamin (**123**) reduced the secretion of the pro-inflammatory cytokine IL-12p40 by dendritic cells (Table 13) [61].

**Figure 14:**
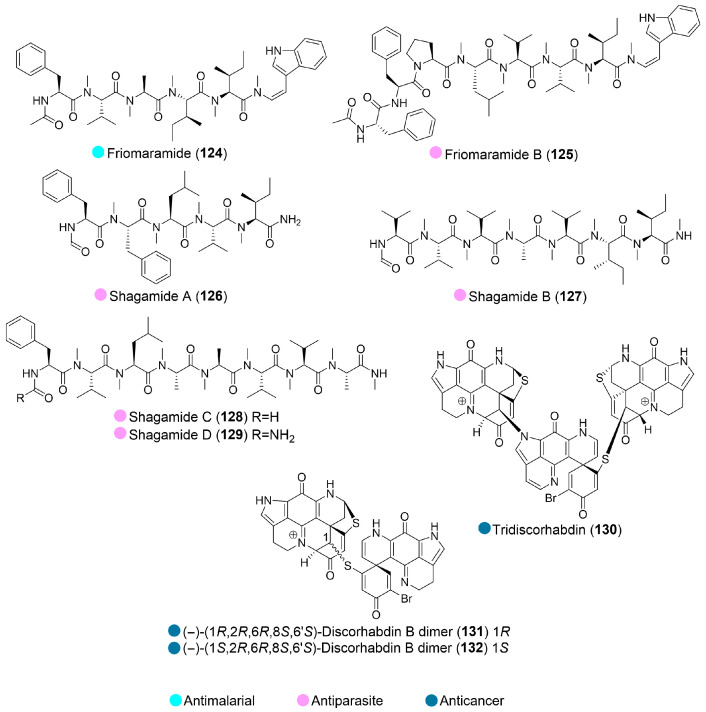
Structures of compounds **124**–**132** and their biological activities.

A new highly modified linear hexapeptide, friomaramide (**124**, Figure 14), was discovered in the Antarctic sponge *Inflatella coelosphaeroides*. At a concentration of 6.1 μM, it suppressed 92% of *Plasmodium falciparum* liver-stage development while exhibiting negligible cytotoxicity toward primary human hepatocytes. Its activity was highly comparable to primaquine, one of the few known liver-stage acting antimalarial drugs, highlighting its potential as a highly selective antimalarial candidate [62].

Five new highly *N*-methylated linear peptides (**125**–**129**, Figure 14) were also isolated from *Inflatella coelosphaeroides* and found to be active against the *Plasmodium falciparum* parasite. All compounds demonstrated promising activity against the drug-resistant strain Dd2. Moreover, friomaramide B (**125**) and shagamides A, C, D (**126**, **128**, and **129**) exhibited viable potential, with activity below 10 μM against multiple strains (Table 14) [63].

A new alkaloid, tridiscorhabdin (**130**, Figure 14), discovered from the deep-sea sponge *Latrunculia biformis* (collected in the southern Weddell Sea, Antarctica), exhibited potent cytotoxic activity against the human colon cancer cell line HCT116. However, it also inhibited the noncancerous human keratinocyte cell line HaCaT, suggesting general cytotoxicity and low selectivity against cancer cells (Table 14) [64].

The Antarctic deep-sea *Latrunculia biformis* also yielded discorhabdin B dimer and its new derivative (**131** and **132**, Figure 14), which showed significant in vitro anticancer activity against the human colon cancer cell line HCT116, with IC_50_ values of 0.16 and 2.01 µM, respectively. However, when tested against the non-cancerous human keratinocyte cell line HaCaT, both compounds showed toxicity, suggesting their low selectivity (Table 14) [65].

**Figure 15:**
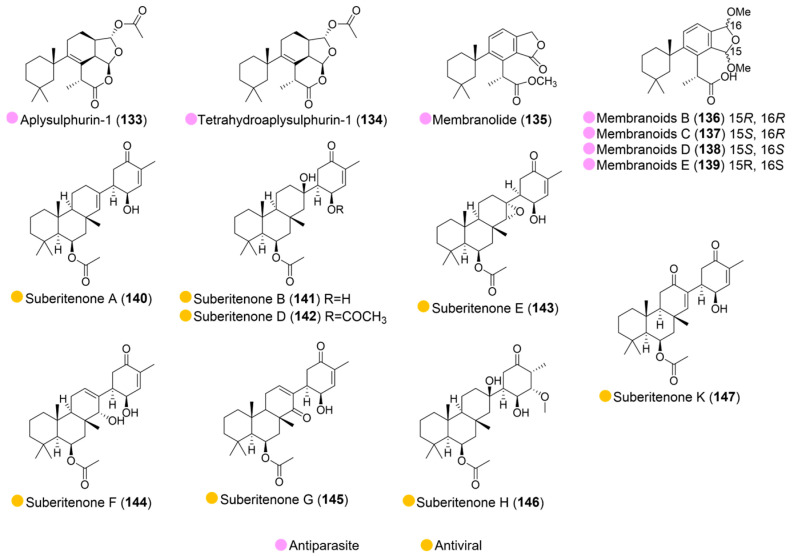
Structures of compounds **133**–**147** and their biological activities.

Seven compounds (**133**–**139**, Figure 15), derived from *Dendrilla antarctica* (collected from various sites around Palmer Station, Antarctica), displayed potent, low micromolar activity against *Leishmania donovani*-infected J774A, with IC_50_ values ranging from 0.8 to 9.7 μM (Table 15). Furthermore, **134**, **136**–**139** demonstrated high selectivity, with no discernible cytotoxicity against uninfected host J774A.1 cells (IC_50_ >133 μM or IC_50_ = 95.0 μM). More interestingly, compounds **133**, **134**, **136**, and **138** were even more active than the positive control, miltefosine—a medication primarily used to treat leishmaniasis [66].

A series of suberitenones (**140**–**147**, Figure 15), derived from the Antarctic sponge *Suberites* sp. (collected within a 3.5 km radius of Palmer Station), was found to be active against respiratory syncytial virus (RSV), with IC_50_ values ranging from 3.2 to 39.8 µM. They also exhibited selectivity for RSV when there were low cytotoxic effects for J774 macrophages or A549 adenocarcinoma cells (selectivity index from 2.4 to 21.1) (Table 15) [67,68].

Antartic and Arctic sponges have yielded high-impact scaffolds, including antibiofilm diterpenes, immunomodulatory and anti-inflammatory diterpenoids, cholinesterase-inhibiting discorhabdins, and potent antiplasmodial peptides. These examples illustrate that sponges concentrate chemically powerful metabolites that target clinically important processes. However, many sponge-derived metabolites suffer from limited selectivity, and it is often unclear whether the true producers are microbial symbionts or the sponge host. Addressing these gaps will require comprehensive sequencing of sponge-associated microbiomes, targeted isolation or single-cell cultivation of producer symbionts, and heterologous expression of BGCs to decouple compound supply from harvesting of slow-growing, vulnerable sponge populations.

## 5. Other Organisms

**Figure 16:**
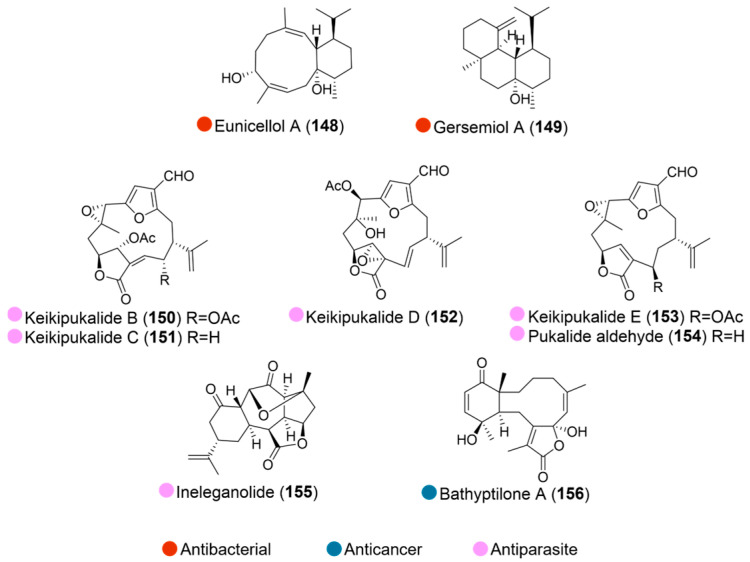
Structures of compounds **148**–**156** and their biological activities.

The Arctic soft coral *Gersemia fruticose*, collected in the Alaskan Arctic Beaufort Sea, yielded two new bioactive diterpenes. Eunicellol A (**148**, Figure 16) displayed moderate, selective antibacterial activity against MRSA, with a minimum inhibitory concentration (MIC_90_) of 24–48 μg/mL, while gersemiol A (**149**, Figure 16) showed marginal activity against MRSA, with a 50% growth inhibition at 48 μg/mL [69].

Six bioactive compounds, including four new furanocembranoid diterpenes, keikipukalides B–E (**150**–**153**, Figure 16), together with two known compounds (**154** and **155**, Figure 16), were isolated from the Antarctic deep-sea octocoral *Plumarella delicatissima* (collected at the Plateau of Fascination NE of the Falkland Islands). They displayed activity against the leishmaniasis parasite, *Leishmania donovani*, with IC_50_ values ranging from 1.9 to 12 μM in the infected macrophage assay, compared to the IC_50_ of 6.2 μM for miltefosine, a drug currently used for the treatment of leishmaniasis (Table 16). No mammalian cytotoxicity was detected in the compounds below 50 μM. However, most of these compounds contain the neurotoxic pharmacophore of lophotoxin, which may prove to be a liability [70].

Bathyptilone A (**156**, Figure 16), a new briarane diterpenes obtained from the Antarctic sea pen *Anthoptilum grandiflorum* (collected in the north of Burdwood Bank, depths of between 662 and 944 m), displayed selective nanomolar cytotoxicity against the neurogenic mammalian cell line Ntera-2 with an IC_50_ value of 29 nM [71].

**Figure 17:**
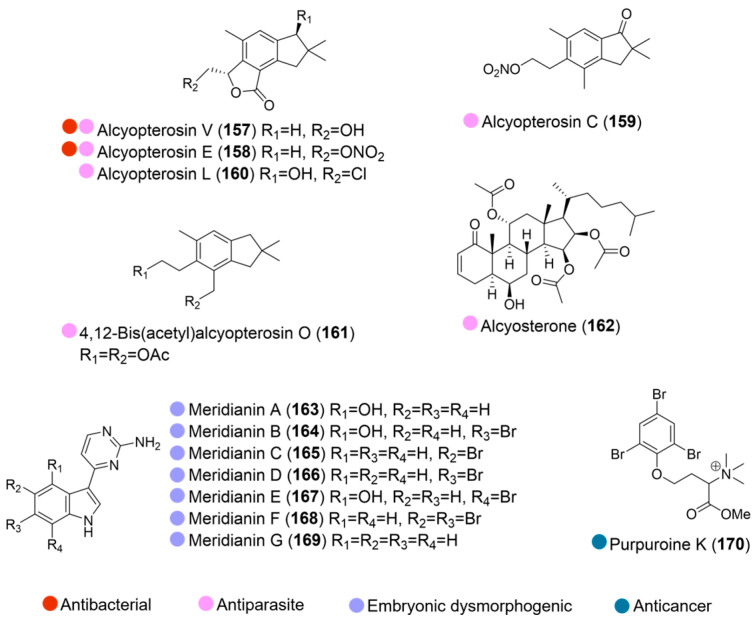
Structure of compounds **157**–**170** and their biological activities.

Deep-water Antarctic octocoral *Alcyonium* sp. yielded six bioactive compounds (**157**–**162**, Figure 17) against *Clostridium difficile* and *Leishmania donovani*, including two new compounds, alcyopterosin V and alcyosterone (**157** and **162**). Most compounds exhibited high potency, with IC_50_ values in the low single-digit micromolar range against *Leishmania donovani*, the causative agent of leishmaniasis (Table 17). Only compounds **157** and **159** were slightly less potent than the control, miltefosine (IC_50_ = 6.2 µM). Furthermore, alcyopterosins E (**158**) and V (**157**) also demonstrated single-digit micromolar activity against *Clostridium difficile*, an intestinal bacterium that can cause severe diarrhea and is increasingly associated with drug resistance (Table 17) [72].

The purified meridianins (**163**–**169**, Figure 17), isolated from *Synoicum* sp. (a tunicate collected at a depth of 200 m near Shag Rocks and South Georgia in Antarctica), exhibited potent bioactivity in a zebrafish developmental assay, inducing distinct embryonic dysmorphology [73].

A new compound, purpuroine K (**170**, Figure 17), isolated from the Arctic echinodermata *Pteraster militaris* (collected at Kvadehuken, Svalbard), showed activity against two acute myeloid leukemia cell lines carrying the FTL3-ITD mutation, MV-4-11 and MOLM-13. In MV-4-11 cells, purpuroine K increased apoptosis and arrested cell cycle in G1/G0, which is a common feature of FLT3 inhibitors [74].

## 6. Future Perspective

The taxonomic and chemical diversity documented in polar regions over the past decade has been constrained by a focus on accessible microbial and fungal lineages, alongside a limited number of sponge taxa, leaving significant portions of the polar biological repertoire unexplored. Future efforts should systematically expand into underexplored taxa, including macroalgae, invertebrates, phytoplankton, zooplankton, and other invertebrates that remain untouched despite their ecological abundance in polar waters. Deliberate sampling across environmental gradients (depth, seasonality, ice cover, salinity) is expected to uncover additional chemical novelty tied to niche-specific ecological pressures.

To bridge the existing knowledge gap, a transition toward mechanism-oriented discovery is essential. The current literature predominantly focuses on isolation and preliminary in vitro bioactivity screening, often leaving a mechanistic void regarding ecological roles, structure–activity relationships, and molecular targets. To address the discrepancy between chemical description and therapeutic application, the discovery pipeline must systematically incorporate target deconvolution, pharmacokinetics, and in vivo validation. Adopting this integrated approach is critical for transforming polar metabolites from preliminary screening hits into robust, clinically credible drug leads.

Despite technological leaps in cultivation, omics, and analytics, these tools are often applied in isolation rather than in synergy. Future bioprospecting should transition toward multidisciplinary frameworks that bridge the gap between initial discovery and sustainable development. By prioritizing scalable production methodologies, researchers can ensure a consistent supply for downstream evaluation while upholding the ecological integrity of fragile polar ecosystems.

Anthropogenic climate change—characterized by rising temperatures, receding sea ice, and shifted primary productivity—is fundamentally reconfiguring polar ecological dynamics and, consequently, the secondary metabolomes of indigenous organisms. Amidst this flux, increasing interest in polar natural products raises concerns about the overexploitation of these highly fragile biospheres. Future endeavors must harmonize discovery with conservation by adopting non-invasive sampling and metabolomics-driven dereplication. Furthermore, leveraging synthetic biology and heterologous expression will be indispensable for decoupling chemical production from the exploitation of fragile biospheres. Ultimately, aligning climate-conscious research with international regulatory frameworks will be critical for the sustainable governance of polar chemical resources.

## 7. Conclusions

From 2015 to 2025, 170 biologically active compounds were isolated from marine and other aquatic organisms inhabiting polar environments. These compounds exhibit diverse, often complex scaffolds and encompass a broad spectrum of biological activities. Antimicrobial, anti-inflammatory, and anticancer activities are most frequently reported, alongside other noteworthy properties such as anti-melanogenic, siderophore, cholinesterase-inhibiting, and embryonic dysmorphogenic effects.

Across taxa, several recurring patterns emerge. Bacterial and fungal producers dominate numerically and yield many compounds with pathogen- or target-selective profiles, including efficacy against multidrug-resistant bacteria, human signaling enzymes, and inflammatory pathways, often amenable to fermentation-based production. In contrast, sponges, tunicates, and other invertebrates frequently provide highly potent metabolites that target clinically relevant processes such as cholinesterase activity, malaria liver stages, biofilm formation, and immune modulation, but these compounds often exhibit limited selectivity and face challenges related to sustainable supply.

Despite the chemical diversity, most studies have focused on cultivable bacteria, fungi, and a limited set of invertebrates (primarily corals and sponges), yet many other unique and potentially potent taxa remain unexplored and warrant further investigation. Moreover, most studies emphasize compound isolation and in vitro bioactivity screening, with limited investigation into mechanisms of action, ecological functions, or SAR, thereby constraining biological interpretation and therapeutic evaluation.

Except for bathyptilone A (**156**) which was active against Ntera-2 cells (IC_50_ = 29 nM), other compounds exhibit their activity only at micromolar concentration, typically falling within low- to high- micromolar IC_50_ or MIC ranges, which must be interpreted with caution in terms of pharmacological relevance. Compounds display sub- (<1 μM) and low- (1–10 μM) micromolar potency with clear selectivity—friomaramide (**124**), which inhibits *P. falciparum* without hepatocyte toxicity, and diterpenoids (**133**–**139**) from *D. antarctica*, which are highly selective against *L. donovani* with no cytotoxicity over the host cell—can be regarded as genuine lead candidates that merit further pharmacological optimization. Compounds exhibiting mid-micromolar activities (10–100 μM) but maintaining high selectivity, such as darwinolide (**114**) and suberitenone F (**147**), may also be considered potential candidates. However, for both groups, follow-up work on selectivity, mechanism, and in vivo efficacy is essential before any conclusions regarding therapeutic usefulness can be made.

By contrast, many reported cytotoxic or antimicrobial effects occur at higher micromolar concentrations and/or are accompanied by comparable toxicity toward non-target mammalian cells, such as tridiscorhabdin (**130**) and discorhabdin B dimers (**131** and **132**). These profiles suggest a narrow therapeutic window and position such compounds more appropriately as chemical probes or starting points for scaffold modification, rather than immediate drug candidates. More broadly, most biological activities compiled in this review are reported from in vitro assay with mid- to high- micromolar active concentration (10–100 μM) against laboratory strains, cell lines, or simple biochemical targets. While these activity levels have limited direct relevance for clinical translation, they represent early-stage discovery hits that primarily serve as chemical probes, mechanistic tools, and sources of structural diversity. Accordingly, when assessing their translational potential, priority should be given to compounds demonstrating target- or cell-selectivity, defined mechanisms of action, chemical stability, and minimal assay artifacts, as mid- to high-micromolar actives frequently capture valuable chemical diversity rather than immediate therapeutic promise.

Overall, the literature compiled in this review underscores the extraordinary chemodiversity inherent in polar aquatic ecosystems, highlighting their immense promise for pharmacological and biotechnological innovation. Simultaneously, however, it exposes systematic taxonomic and methodological disparities that continue to shape and limit our current understanding of this unique chemical space. Crucially, without systematic evaluation of pharmacokinetics, toxicity, and in vivo efficacy, the translational potential of polar natural products will remain largely speculative, underscoring the need to move beyond descriptive chemical diversity toward meaningful therapeutic development.

## Figures and Tables

**Table 1:** In vitro biological activities of compounds **1**–**13**.

Source	Compounds	Bioactivity	Reference
*Vibrio splendidus* (Antarctica)	**1**	Cytotoxicity (HT-29, GXF 251, LXFL 529, LXFA 629, MAXF 401, MEXF 462, OVXF 899, PAXF 1657, 22Rv1, RXF 486, UXF 1138); IC_50_ = 0.50–7.82 µM	[10]
		Antimicrobial activity (*B. subtilis*, *S. aureus*, *S. viridochromogenes*, *E. coli*, *C. albicans*, *M. miehei*, *P. ultimum*); Inhibition zone diameter 12–16 mm	
	**2**	Antimicrobial activity (*B. subtilis*, *S. aureus*, *E. coli, C. albicans*, *M. miehei*); Inhibition zone diameter 12–14 mm	
	**3**	Cytotoxicity against human cancer cell lines (HT-29, GXF 251, LXFL 529, LXFA 629, MAXF 401, MEXF 462, OVXF 899, PAXF 1657, 22Rv1, RXF 486, UXF 1138); IC_50_ = 16.23–26.57 µM	
		Antimicrobial activity (*B. subtilis*, *S. aureus*, *E. coli, C. albicans*, *M. miehei*); Inhibition zone diameter 12–15 mm	
	**4**	Antimicrobial activity (*B. subtilis*, *S. viridochromogenes*, *E. coli*); Inhibition zone diameter 12–20 mm	
	**5**	Antimicrobial activity (*B. subtilis*, *S. viridochromogenes*, *E. coli*); Inhibition zone diameter 12–18 mm	
	**6**	Antimicrobial activity (*B. subtilis*, *S. viridochromogenes*, *E. coli*); Inhibition zone diameter 12–18 mm	
	**7**	Antimicrobial activity (*B. subtilis*, *S. aureus*, *E. coli, C. albicans*, *M. miehei, P. ultimum*); Inhibition zone diameter 10–15 mm	
	**8**	Antimicrobial activity (*B. subtilis*, *S. aureus*, *S. viridochromogenes*, *E. coli*, *C. albicans*, *M. miehei*); Inhibition zone diameter 10–20 mm	
	**9**	Antimicrobial activity (*B. subtilis*, *S. aureus*, *E. coli*); Inhibition zone diameter 10–17 mm	
	**10**	Antimicrobial activity (*B. subtilis*, *S. aureus*, *E. coli*); Inhibition zone diameter 10–17 mm	
	**11**	Cytotoxicity against human cancer cell lines (HT-29, GXF 251, LXFL 529, LXFA 629, MAXF 401, MEXF 462, OVXF 899, PAXF 1657, 22Rv1, RXF 486, UXF 1138); IC_50_ = 7.91–50.15 µM	
		Antimicrobial activity (*B. subtilis*, *E. coli*); Inhibition zone diameter 25–20 mm	
	**12**	Cytotoxicity against human cancer cell lines (HT-29, GXF 251, LXFL 529, LXFA 629, MAXF 401, MEXF 462, OVXF 899, PAXF 1657, 22Rv1, RXF 486, UXF 1138); IC_50_ = 8.21–12.58 µM	
	**13**	Cytotoxicity against human cancer cell lines (HT-29, GXF 251, LXFL 529, LXFA 629, MAXF 401, MEXF 462, OVXF 899, PAXF 1657, 22Rv1, RXF 486, UXF 1138); IC_50_ = 7.08–53.74 µM	

**Table 2:** In vitro biological activities of compounds **14**–**21**.

Source	Compounds	Bioactivity	Reference
*Bacillus amyloliquefaciens* Pc3 (Antarctica)	**14**	Antifungal activity (*T. viride*, *C. gloeosporioides*, *S. sclerotiorum*, *F. oxysporum*, *A. longipes*, *R. solani* Kühn, *P. variotii*); MIC = 3.125, 6.25, 6.25, 3.125, 3.125, 6.25, 3.125 μg/disc	[11]
*Pseudoalteromonas haloplanktis* TAC125 (Antarctica)	**15**	Anti-biofilm activity (*S. epidermidis*) at 60 μg/mL	[12]
*Pseudomonas* sp. M10B774 (Arctic)	**16**	Antimicrobial activity (*E. faecalis*, *S. aureus*, *S. agalactiae*) at 50 µM Anti-biofilm (*S. epidermidis*) at 50 µM	[13]
	**17**	Antimicrobial activity (*E. faecalis*, *S. aureus*, *S. agalactiae*) at 50 µM Anti-biofilm activity (*S. epidermidis*) at 50 µM Cytotoxicity (A2508, MRC5) at 150 µM	
	**18**	Antimicrobial activity (*E. faecalis*, *S. aureus*, *S. agalactiae*) at 50 μM Anti-biofilm activity (*S. epidermidis*) at 100 µM	
	**19**	Antimicrobial activity (*E. faecalis*, *S. aureus*, *S. agalactiae*) at 50 μM Anti-biofilm activity (*S. epidermidis*) at 100 µM Cytotoxicity (A2508, MRC5) at 150 µM	
	**20**	Antimicrobial activity (*E. faecalis*, *S. aureus*, *S. agalactiae*) at 50 μM Anti-biofilm activity (*S. epidermidis*) at 100 µM	
	**21**	Antimicrobial activity (*E. faecalis*, *S. aureus*, *S. agalactiae*) at 50 μM Anti-biofilm activity (*S. epidermidis*) at 100 µM Cytotoxicity (A2508, MRC5) at 100 and 150 µM	

**Table 3:** In vitro biological activities of compounds **22**–**29**.

Source	Compounds	Bioactivity	Reference
*Aequorivita* sp. (Antarctica)	**22**–**25**	Antimicrobial activity (MRSA); IC_50_ = 22–145 µg/mL	[14]
*Streptomyces* sp. (Antarctica)	**26**	Cytotoxicity (H1299, A549, HCT116, PC3, Mia-paca2, ASPC1, HepG2, HeLa, U87, Cal62, CHL-1, and SK-Mel28) at 20 µg/mL; Induced G1 cell cycle arrest (A549, H1299, U87) at 10 µg/mL	[15]
*Variovorax* sp. (Arctic)	**27**	Antimicrobial activity (MRSA, vancomycin-resistant *Enterococcus*, *S. warneri*, and *P. vulgaris*); IC_50_ = 35, 11, 28, 14 μM,	[16]
*Streptomyces* sp. OUCMDZ-4348 (Antarctica)	**28**	Cytotoxicity (N87 cells); IC_50_ = 9.8 μM	[17]
Co-culture of *Serratia* sp. and *Shewanella* sp. (Arctic)	**29**	Antimicrobial activity (*S. aureus*); MIC = 25 μM Cytotoxicity (A2058, MRC5) at 10 and 5 μM Anti-biofilm activity (*S. epidermidis*) at 200 μM	[18]

**Table 4:** In vitro biological activities of compounds **30**–**36**.

Species	Compounds	Bioactivity	Reference
*Nocardiopsis aegyptia* HDN19-252 (Antarctica)	**30**	Antimicrobial activity (MRCNS, *B. subtilis*, *B. cereus*, *E. coli*, *M. Phlei*); MIC = 6.2 µM Antimicrobial activity (*P. species*); MIC = 12.5 µM	[19]
	**31**	Antimicrobial activity (MRCNS, *B. subtilis*, *P. species*, *B. cereus*, *E. coli*); MIC = 6.2 µM Antimicrobial activity (*M. Phlei*); MIC = 3.1 µM	
*Streptomyces* sp. SCO-736 (Antarctica)	**32**	Anti-melanogenic activity at 50 ppm	[20]
*Nocardiopsis* sp. LX-1 (Antarctica)	**33**	Antimicrobial activity (*F. fujikuroi*); inhibition zone radius 6.5 mm at 100 µM Antimicrobial activity (*A. hydrophila*); MIC = 100 µM	[21]
	**34**	Antimicrobial activity (*A. hydrophila*, *D. chrysanthemi*, *C. terrigena*, *X. citri pv. Malvacearum*, *C. albicans*); MIC = 100, 100, 100, 25, 100 µM	
	**35**	Antimicrobial activity (*D. citri*), inhibition zone radius 5.3 mm at 100 µM	
	**36**	Antimicrobial activity (*D. citri*), inhibition zone radius 14 mm at 100 µM	

**Table 5:** In vitro biological activities of compounds **37**–**43**.

Species	Compounds	Bioactivity	Reference
*Streptomyces somaliensis* 1107 (Arctic)	**37**	Anti-inflammatory activity without cytotoxicity Inhibited production of IL-6 (IC_50_ = 5.76 μM) and TNF-α (IC_50_ = 0.18 μM) in LPS-stimulated RAW264.7	[22]
*Streptomyces* sp. DSS69 (Antarctica)	**38**	Antimicrobial activity (*S. aureus*, MRSA, MRSE, *E. faecalis*, *M. luteus*, *B. altitudinis*, *L. monocytogenes*, *C. albicans*); MICs = 0.10–3.33 µg/mL Cytotoxicity (HL-60, HepG2, U-87MG, HCT116); IC_50_ = 2.07–11.50 µM	[23]
*Bacillus amyloliquefaciens* SCSIO 41392 (Arctic)	**39** and **40**	Inhibited PQS QS system at 50 µg/mL Inhibited pyocyanin production at 50 µg/mL	[24]
	**41**–**43**	Inhibited pyoverdine production at 50 µg/mL **43**: Anti-biofilm activity (*P. aeruginosa*) at 50 µg/mL	

**Table 6:** In vitro biological activities of compounds **44**–**50**.

Source	Compounds	Bioactivity	Reference
Lindgomycetaceae strain (Arctic and Baltic Sea)	**44**	Antimicrobial activity (*B. subtilis*, *X. campestris*, *S. epidermidis*, *S. aureus*, MRSA, *P. acnes*); IC_50_ = 2.2, 17.8, 4.6, 2.7, 5.1, 4.7 μM Antifungal activity (*C. albicans*, *S. tritici*); IC_50_ = 5.7, 5.1 μM	[25]
	**45**	Antimicrobial activity (*B. subtilis*, *X. campestris*, *S. epidermidis*, *S. aureus*, MRSA, *P. acnes*); IC_50_ = 3.4, 14.8, 6.3, 2.9, 3.2, 2.8 μM Antimicrobial activity (*C. albicans*, *S. tritici*); IC_50_ = 8.0, 10.0 μM	
*Penicillium funiculosum* GWT2-24 (Antarctica)	**46**–**48**	Antimicrobial activity (H1N1); IC_50_ = 21, 55, 57 μM	[26]
*Penicillium* sp. HDN14-431 (Antarctica)	**49**	Antimicrobial activity (*P. vulgarisi*); MIC = 22.5 μM	[27]
*Penicillium* sp. S-1-18 (Antarctica)	**50**	Inhibited tyrosine phosphatase 1B, IC_50_ = 27.4 μM	[28]

**Table 7:** In vitro biological activities of compounds **51**–**59**.

Source	Compounds	Bioactivity	Reference
*Aspergillus sydowii* SP-1 (Antarctica)	**51**–**54**	**51**: Antimicrobial activity (*S. aureus*, MRSA, *S. epidermidis*, MRSE); MIC = 8, 32, 4, 16 μg/mL	[29]
		**52**: Antimicrobial activity (*S. aureus*, MRSA, *S. epidermidis*, MRSE); MIC = 0.25, 1.00, 0.12, 0.5 μg/mL	
		**53**: Antimicrobial activity (*S. aureus*, MRSA, *S. epidermidis*, MRSE); MIC = 0.5, 1.0, 0.25, 0.5 μg/mL	
		**54**: Antimicrobial activity (*S. aureus*, MRSA, *S. epidermidis*, MRSE); MIC = 0.5, 1.0, 0.25, 0.5 μg/mL	
*Penicillium citreonigrum* SP-6 (Antarctica)	**55** and **56**	Cytotoxicity (HCT116); IC_50_ = 26.7 and 46.3 μM	[30]
*Penicillium crustosum* HDN153086 (Antarctica)	**57**	Cytotoxicity (K562); IC_50_ = 12.7 μM	[31]
*Aspergillus insulicola* HDN151418 (Antarctica)	**58** and **59**	**58**: Antimicrobial activity (*B. cereus*, *P. species*, *M. phlei*, *E. tarda, B. subtilis*, MRCNS, MRSA, *V. parahemolyticus*); MIC = 3.13, 3.13, 3.13, 1.56, 6.25, 12.5, 25.0 μM	[32]
		**59**: Antimicrobial activity (*B. cereus*, *P. species*, *M. phlei*, *E. tarda, B. subtilis*, MRCNS, MRSA, *V. parahemolyticus*); MIC = 6.25, 6.25, 12.5, 1.56, 12.5, 25.0, 25.0, 6.25 μM	

**Table 8:** In vitro biological activities of compounds **60**–**71**.

Source	Compounds	Bioactivity	Reference
*Penicillium glabrum* SF-7123 (Antarctica)	**60**–**62**	Inhibited NO production; IC_50_ = 2.7, 28.1, 10.6 μM Inhibited PGE_2_ production; IC_50_ = 3.2, 25.2, 32.3 μM	[33]
*Penicillium* sp. HDN151272 (Antarctica)	**63**	Antimicrobial activity (MRCNS, *B. cereus*, *P. aeruginosa*, *M. phlei*). MIC = 6.25, 12.5, 1.56, 3.13 µg/mL	[34]
	**64**	Antimicrobial activity (*B. subtilis,* MRCNS, *B. cereus*, *P. aeruginosa*, *M. phlei*). MIC = 12.5, 6.25, 25.0, 6.25, 6.25 µg/mL	
*Digitatispora marina* (Antarctica)	**65**	Cytotoxicity (A2058); ~50% survival at 50 µM	[35]
*Aspergillus candidus* HDN15-152 (Antarctica)	**66**	Antimicrobial activity (H1N1); IC_50_ = 26.0 μM	[36]
	**67**	Cytotoxicity (HL-60); IC_50_ = 7.8 μM	
*Penicillium echinulatum* (Antarctica)	**68** and **69**	UV absorption (UVB, UVA-II range), λ_c_ = 335 and 334 nm	[37]
*Talaromyces* sp. HDN1820200 (Antarctica)	**70**	Antimicrobial activity (*P.mirabilis*); MIC = 12.5 μM	[38]
	**71**	Antimicrobial activity (*P.mirabilis*, *V. parahemolyticus*); MIC = 3.13, 6.13 μM	

**Table 9:** In vitro biological activities of compounds **72**–**80**.

Source	Compounds	Bioactivity	Reference
Species belong to Lulworthiaceae (Arctic)	**72**	Antimicrobial activity (*S. aureus* N315, *S. aureus* 85/2082, *S. aureus* NCTC 10442, *S. aureus* WIS [WBG8318], *S. aureus* IHT 99040, *S. aureus* ATCC^®^ 25923, *S. agalactiae* ATCC^®^ 12386); MIC = 1.56, 3.13, 3.13, 6.25, 3.13, 6.25, 12.5 μg/mL Cytotoxicity (A2058, MRC5, HepG2); IC_50_ = 15.5, 32.0, 27.0 µg/mL	[39,40]
*Tolypocladium* sp. SCSIO 40433 (Arctic)	**73**	Inhibited tyrosinase activity (20 and 40 µM).	[41]
*Alternaria* sp. HDN19-690 (Antarctica)	**74**	Antimicrobial activity (MRCNS, *B. subtilis*, *P. mirabilis*, *B. cereus*, *E. coli*, and *M. phlei*); MIC = 3.13, 3.13, 12.5, 3.13, 12.5, 6.25 µM	[42]
	**75**	Antimicrobial activity (MRCNS, *B. subtilis*, *P. mirabilis*, *B. cereus*, *E. coli*, and *M. phlei*); MIC = 12.5, 12.5, 25.0, 12.5, 6.25, 25.0 µM	
	**76**	Antimicrobial activity (MRCNS, *B. subtilis*, *P. mirabilis*, *B. cereus*, *E. coli*, and *M. phlei*); MIC = 12.5, 12.5, 25.0, 12.5, 12.5, 12.5 µM	
	**77**	Antimicrobial activity (MRCNS, *B. subtilis*, *B. cereus*, *E. coli*, and *M. phlei*); MIC = 25.0, 50.0, 25.0, 25.0, 25.0 µM	
*Pseudogymnoascus* sp. HDN17-933 (Antarctica)	**78**	Inhibited nicotinic acetylcholine receptor subtypes (>70% inhibition at 100 µM)	[43]
*Aspergillus insulicola* HDN151418 (Antarctica)	**79**	Cytotoxicity (MAD-MB-231, AsPC-1, PANC-1); IC_50_ = 11.7, 2.7, 4.6 µM	[44]
	**80**	Cytotoxicity (MAD-MB-231, AsPC-1, PANC-1); IC_50_ = 22.9, 2.3, 4.2 µM	

**Table 10:** In vitro and in vivo biological activities of compounds **86**–**92**.

Source	Compounds	Bioactivity	Reference
*Penicillium palitans* (Antarctica)	**86**	Phytotoxicity (*Agrostis stolonifera*, 100% at 1 mg/mL, *Lemna paucicostata*, IC_50_ = 57 µM) Antimicrobial activity (*C. fragariae*); inhibition zones 12.0 mm at 100 μg/spot and 6.3 mm at 10 μg/spot; IC_50_ = 0.3 μM	[49]
*Pseudogymnoascus* sp. HDN17-895 (Antarctica)	**87**–**89**	Anti-inflammatory in vitro; IC_50_ = 1.13–6.96 μM **87**: Reduced the concentration of IL-1β in mice serum (50 mg/kg)	[50]
*Aspergillus candidus* HDN15-152 (Antarctica)	**90**–**92**	**90**: Cytotoxicity (NCI-H446, NCI-H446/EP, L-02); IC_50_ = 9.77, 12.38 μM **91**: Cytotoxicity (NCI-H446/EP); IC_50_ = 16.5 μM **92**: Antimicrobial activity (Influenza A virus A/PR/8/34(H1N1) strain); IC_50_ = 39.2 μM, ribavirin IC_50_ = 37.2 μM	[51]

**Table 11:** In vitro biological activities of compounds **93**–**102**.

Source	Compounds	Bioactivity	Reference
*Simplicillium lamellicola* (Arctic)	**93**–**95**	Inhibited AR signaling pathway at 25 μM	[52]
*Aspergillus japonicas* (Arctic)	**96**–**99**	Antioxidant activity (50 μg, 100 μM) **96**: Anti-inflammatory activity; EC_50_ = 18.77 μM **97**: Anti-inflammatory activity; EC_50_ = 72.14 μM, no cytotoxicity	[53]
*Uzbekistanica storfjordensis* sp. nov (Arctic)	**100**–**102**	Antimicrobial activity (*S. agalactiae*); MIC = 86, 66, 64 μM	[54]

**Table 12:** In vitro biological activities of compound **113**.

Source	Compounds	Bioactivity	Reference
*Aspergillus* sp. (strain SF7367) (Antarctica)	**113**	Anti-inflammatory activity; Inhibited excessive nitrite, TNFα, IL-6 at 40, 10, 40 µM Suppressed iNOS expression at 10 µM, NF-κB activation at 20–40 µM	[57]

**Table 13:** In vitro and in vivo biological activities of compounds **114**–**123**.

Source	Compounds	Bioactivity	Reference
*Dendrilla membranosa* (Antarctica)	**114**	Anti-biofilm activity (MRSA); IC_50_ = 33.2 µM, MIC (on planktonic MRSA) = 132.9 μM Low cytotoxicity (mammalian cell); IC_50_ = 73.4 μM	[58]
	**115**	Cytotoxicity (PBMC) at 10 μM In vivo anti-inflammatory activity (in murine dermatitis model) at 1.0 μM/cm^2^	[59]
Antarctic *Latrunculia* spp. (Antarctica)	**116**–**119**	**116**: Cholinesterase-inhibiting activity (eeAChE, hAChE, BChE); *K_i_* = 1.6, 56.2, 5.0 μM **117**: Cholinesterase-inhibiting activity (eeAChE, hAChE, BChE); *K_i_* = 3.5, 9.8, 17.5 μM **118**: Cholinesterase-inhibiting activity (eeAChE, hAChE, BChE); *K_i_* = 1.9, 22.8, 76.0 μM **119**: Cholinesterase-inhibiting activity (eeAChE); *K_i_* = 15.0 μM	[60]
*Geodia barretti* (Arctic)	**120**–**123**	**120**: Anti-inflammatory activity at 10 μg/mL; inhibited 29% IL-12p40 **121**: Anti-inflammatory activity at 10 μg/mL; inhibited 13% IL-12p40 **122**: Anti-inflammatory activity at 10 μg/mL; inhibited >50% IL-12p40, IL-10 **123**: Anti-inflammatory activity at 10 μg/mL; inhibited 32% IL-12p40	[61]

**Table 14:** In vitro biological activities of compounds **125**–**132**.

Source	Compounds	Bioactivity	Reference
*Inflatella coelosphaeroides* (Antarctica)	**125**–**129**	**125**: Antiparasite (*P. falciparum* strains NF54, Dd2, 3D7); IC_50_ = 3.93, 3.30, 8.65 μM **126**: Antiparasite (*P. falciparum* strains NF54, Dd2, 3D7); IC_50_ = 2.61, 1.72, 2.10 μM **127**: Antiparasite (*P. falciparum* strains Dd2,); IC_50_ = 4.37 μM **128**: Antiparasite (*P. falciparum* strains NF54, Dd2, 3D7); IC_50_ = 3.73, 1.07, 2.52 μM **129**: Antiparasite (*P. falciparum* strains NF54, Dd2); IC_50_ = 3.05, 2.02 μM	[63]
*Latrunculia biformis* (Antarctica)	**130**	Cytotoxicity (HCT116, HaCaT); IC_50_ = 0.31, 0.92 μM	[64]
	**131**	Cytotoxicity (HCT116, HaCaT); IC_50_ = 0.16, 0.56 µM	[65]
	**132**	Cytotoxicity (HCT116, HaCaT); IC_50_ = 2.01, 4.69 µM	

**Table 15:** In vitro biological activities of compounds **133**–**147**.

Source	Compounds	Bioactivity	Reference
*Dendrilla antarctica* (Antarctica)	**133**–**139**	Antiparasite (*L. donovani*); IC_50_ = 3.1, 3.5, 9.7, 0.8, 6.5, 1.4, 6.6 μM **134**, **136**–**138**: High selectivity, no cytotoxicity (J774A.1 cells)	[66]
*Suberites* sp. (Antarctica)	**140**–**147**	**140**: Antiviral activity (RSV); IC_50_ = 7.8 µM. Selectivity Index 4.3 **141**: Antiviral activity (RSV); IC_50_ = 3.2 µM. Selectivity Index 21.1 **142**: Antiviral activity (RSV); IC_50_ = 15.0 µM. Selectivity Index 2.4 **143**: Antiviral activity (RSV); IC_50_ = 20.5 µM. Selectivity Index 4.3 **144**: Antiviral activity (RSV); IC_50_ = 9.8 µM. Selectivity Index 3.7 **145**: Antiviral activity (RSV); IC_50_ = 11.0 µM. Selectivity Index 3.7 **146**: Antiviral activity (RSV); IC_50_ = 10.9 µM. Selectivity Index 4.7 **147**: Antiviral activity (RSV); IC_50_ = 39.8 µM. No cytotoxicity	[67,68]

**Table 16:** In vitro biological activities of compounds **150**–**155**.

Source	Compounds	Bioactivity	Reference
*Plumarella delicatissima* (Antarctica)	**150**–**155**	**150**: Antiparasite (*L. donovani*); IC_50_ = 8.5 μM **151**: Antiparasite (*L. donovani*); IC_50_ = 8.8 μM **152**: Antiparasite (*L. donovani*); IC_50_ = 12.0 μM **153**: Antiparasite (*L. donovani*); IC_50_ = 8.8 μM **154**: Antiparasite (*L. donovani*); IC_50_ = 1.9 μM **155**: Antiparasite (*L. donovani*); IC_50_ = 4.4 μM	[70]

**Table 17:** In vitro biological activities of compounds **157**–**162**.

Source	Compounds	Bioactivity	Reference
*Alcyonium* sp. (Antarctica)	**157**–**162**	**157**: Antiparasite (*L. donovani*); IC_50_ = 7.0 µM. Antimicrobial (*C. difficile*); MIC = 8.1 µM **158**: Antiparasite (*L. donovani*); IC_50_ = 3.1 µM. Antimicrobial (*C. difficile*); MIC = 6.9 µM **159**: Antiparasite (*L. donovani*); IC_50_ = 13 µM **160**: Antiparasite (*L. donovani*); IC_50_ = 2.4 µM **161**: Antiparasite (*L. donovani*); IC_50_ = 1.2 µM **162**: Antiparasite (*L. donovani*); IC_50_ = 1.5 µM	[72]

## Data Availability

No new data were created or analyzed in this study. Data sharing is not applicable to this article.
